# Ag-Cu nanoalloyed film as a high-performance cathode electrocatalytic material for zinc-air battery

**DOI:** 10.1186/s11671-015-0900-9

**Published:** 2015-04-23

**Authors:** Yimin Lei, Fuyi Chen, Yachao Jin, Zongwen Liu

**Affiliations:** State Key Laboratory of Solidification Processing, Northwestern Polytechnical University, 127 Youyi Road, Xi’an, Shaanxi 710072 China; School of Chemical and Biomolecular Engineering, University of Sydney, Chemical Engineering Building, Sydney, NSW 2006 Australia

**Keywords:** Pulsed laser deposition, Alloyed Ag-Cu, Electrocatalyst, Zinc-air battery

## Abstract

A novel Ag_50_Cu_50_ film electrocatalyst for oxygen reduction reaction (ORR) was prepared by pulsed laser deposition (PLD) method. The electrocatalyst actually is Ag-Cu alloyed nanoparticles embedded in amorphous Cu film, based on transmission electron microscopy (TEM) characterization. The rotating disk electrode (RDE) measurements provide evidence that the ORR proceed *via* a four-electron pathway on the electrocatalysts in alkaline solution. And it is much more efficient than pure Ag catalyst. The catalytic layer has maximum power density of 67 mW cm^−2^ and an acceptable cell voltage at 0.863 V when current densities increased up to 100 mA cm^−2^ in the Ag_50_Cu_50_-based primary zinc-air battery. The resulting rechargeable zinc-air battery exhibits low charge-discharge voltage polarization of 1.1 V at 20 mAcm^−2^ and high durability over 100 cycles in natural air.

## Background

Metal-air batteries have attracted a lot of attention as a possible energy storage solution for decades [1]. Among various metal-air batteries, zinc-air battery holds the greatest promise for future energy applications [2], owing to its high power density, safety, and economic viability and the abundant zinc reserve in earth [3-5]. The key point for development of zinc-air battery is finding high-performance oxygen reduction reaction (ORR) catalysts. Precious metals, like Pt, are usually used as ORR catalysts [6,7]. In general, Pt-based catalysts can be prepared *via* various routes, such as electrochemical deposition [8], chemical vapor deposition [9,10], and facile hydrothermal method [11]. Since the catalyst utilization in the fuel cell is determined mainly by the contact surface area of catalyst with electrolyte, the reduction of the thickness of catalytic layer can result in an improvement of the catalyst utilization and reduction of the fuel cell cost [12]. Therefore, pulse laser deposition (PLD) is also used popularly in the field [13-15], because the approach is a feasible way to control the thickness of catalyst layer. Moreover, compared to aforementioned chemical preparations, PLD method also owns high repeatability and stability in process, making it to be a suitable route to obtain electrocatalyst with film state.

Although Pt-based film catalyst has already obtained progress *via* PLD method [15], it is still expected to further decrease the cost of ORR catalyst. Therefore, the 3d transition metal oxides [16-19], silver [20-27], and its related alloy with transition metals, such as Ag-Co and Ag-Mn [28-31], which consume less cost than Pt were investigated extensively in an alkaline environment. Beyond that, Ag-Cu alloyed materials also might be suitable electrocatalyst for reasons. Theoretical calculations indicate that Ag-Cu alloyed nanoparticles exhibit strong adsorption energies and low activation-energy barriers [32,33]. Meanwhile, Ag and Cu own the same facet-center cubic structure and similar cell parameters. Synthesis of Ag-Cu alloyed catalyst is supposed to be easier than other types of Ag alloy [34]. It is reasonable to believe that Ag-Cu nanoalloy could become a new generation of catalysts. However, successful synthesis of real Ag-Cu alloyed catalysts in nanoscale has seldom been reported aside from some Ag-Cu heterostructures which do not have alloy state [35,36].

Based on the aforementioned background, herein, we expect PLD method can be applied to prepare Ag-Cu alloyed catalyst film, which not only does greatly reduce the catalytic cost but also can obtain effective ORR catalytic activity. In this work, we demonstrate a design of Ag-Cu alloyed film electrocatalyst synthesized *via* pulsed laser deposition (PLD). The ORR catalytic property of the as-prepared Ag-Cu electrocatalyst has been tested and compared to commercial Pt/C, Ir/C, and Ag film catalysts. Both of the resulting primary and rechargeable zinc-air battery show good performance in natural air.

## Methods

### Synthesis of Ag_50_Cu_50_ film electrocatalyst

Ag-Cu alloyed catalyst was prepared by PLD method in a vacuum chamber with a pressure of 2 × 10^−4^ Pa. The target of Ag-Cu alloy with atomic ratio of 50:50 was irradiated with a nanosecond Q-switched Nd:YAG laser beam (EKSPLA, Lithuania). The wavelength was set to be 266 nm, and the pulse duration was ranging from 3 to 6 ns. The laser beam diameter was around 1 mm, with an energy density of 200 mJ/pulse. Both target and substrate (nickel foam) rotated at a speed of 5 rpm during deposition, and target was irradiated for 2 min at 10 Hz to clear away the oxide on the surface before deposition. The laser was operated at the frequency of 10 Hz. The deposition time is set as 90 min. The as-prepared product is Ag_50_Cu_50_ catalyst.

### Electrochemical measurements

The ORR activities of Ag_50_Cu_50_ catalyst were studied at room temperature *via* measuring rotating disk electrode (RDE) polarization curves. The experiments were performed with a classic three-electrode cell containing a saturated calomel electrode (SCE) as reference electrode, a Pt counter electrode, and Ag-Cu catalyst supported on nickel foam as the working electrode in the CHI660C electrochemical workstation. The electrolyte was 0.1 M KOH aqueous solution. The experiments were performed over the potential range of 0 to −0.8 V at a scanning rate of 10 mV s^−1^. All potentials reported in this work were converted from the SCE to the RHE scale using *E*(RHE) = *E*(SCE) + 0.991 V in 0.1 M KOH. The rotation rates were controlled at 400, 800, 1,200, and 1,600 rpm. The working area for oxygen diffusion in the air electrode was 0.785 cm^2^.

### Performance test of zinc-air battery

The catalyst layer was made by the direct deposition of Ag-Cu alloyed catalyst onto the nickel foam current collector. The air cathode was then assembled by the catalyst layer and the gas diffusion layer prepared by mixing acetylene black and 60% PTFE with a mass ratio of 1:2. A zinc-air battery was then fabricated by the prepared air cathode and pure zinc plate anode and 6 M KOH electrolytes with 0.2 M zinc acetate solution. The cell voltage polarization, power density curves, and specific capacity for the primary zinc-air battery have been tested. The charge and discharge characteristic and the cycle performance of the resulting zinc-air battery were tested by the BTS-9000 battery test system in a homemade metal-air battery model in air where air was introduced from the backside of the gas diffusion layer.

### Material characterization on AgCu alloyed catalysts

Ag_50_Cu_50_ catalyst was investigated through comprehensive characterization with scanning electron microscope (SEM), transmission electron microscope (TEM), high-resolution TEM, and high-angle annular dark field (HAADF) carried out on a JEM-2200FS TEM (JEOL Ltd., Akishima-shi, Japan). An empty Ni grid was placed in PLD instrument with nickel foam to directly obtain TEM sample for plain viewing observation.

## Results and discussion

### Characterization on Ag_50_Cu_50_ catalyst

**Figure 1:**
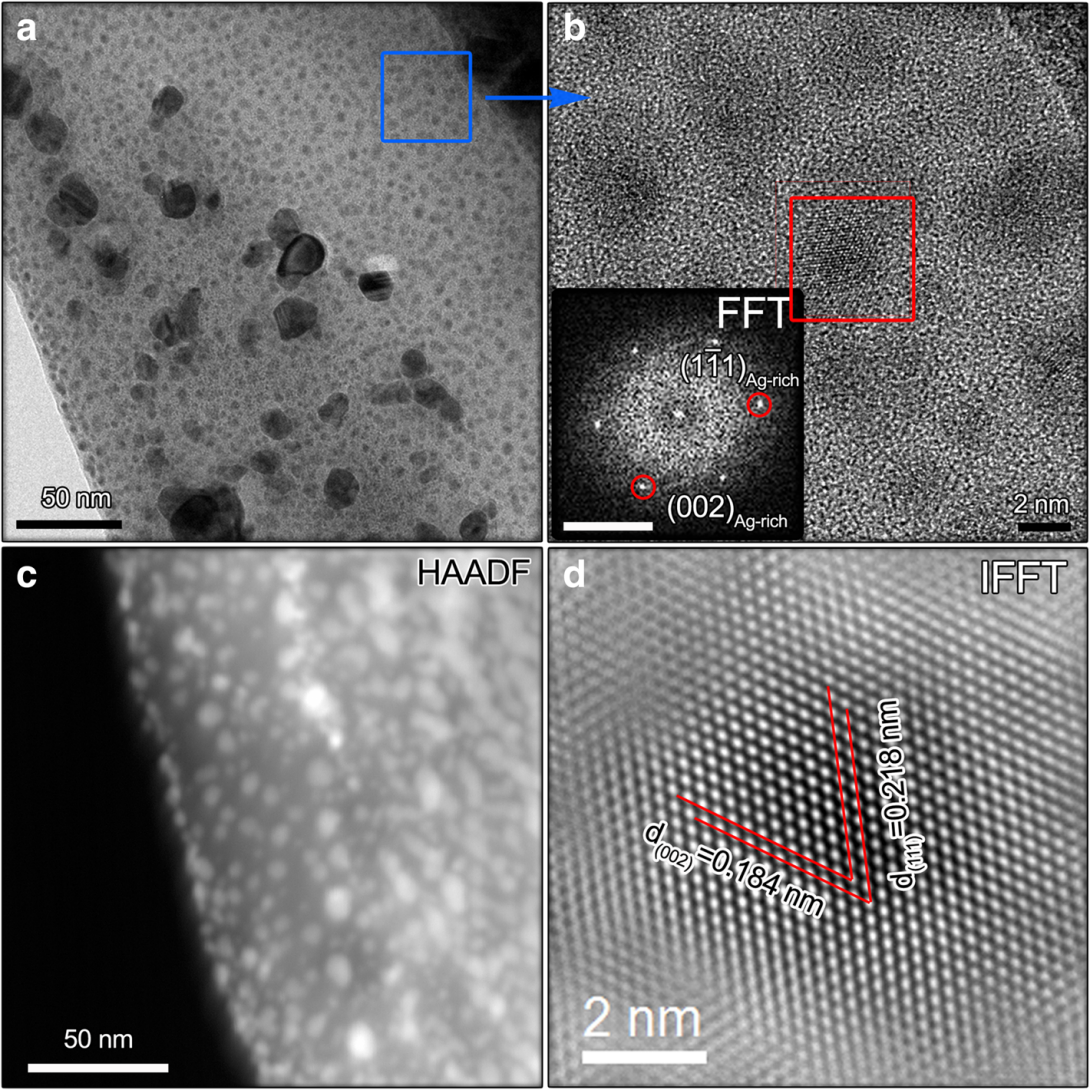
TEM and HAADF characterization of Ag_50_Cu_50_ catalysts. **(a)** Bright field image, **(b)** HRTEM, **(c)** HAADF result, and **(d)** IFFT image. The scale bar for the inset FFT image in **(b)** is 5 nm^−1^.

Figure 1 shows series of TEM analysis on Ag_50_Cu_50_ catalyst. According to Figure 1a, plenty of nanoparticles distribute in a continuous film. The tiny nanoparticles with size under 5 nm dominate the film. Magnifying the blue rectangle area, the obtained HRTEM is shown in Figure 1b. It can be seen that they display two different states: few are amorphous, and the left are with crystallized state.

Fast Fourier transform (FFT) analysis has been performed on particle marked with red rectangle in Figure 1b. Based on the inset FFT image, it is believed that the particle has FCC structure with zone axis of [110]. The corresponding IFFT image is shown in Figure 1d. The d-spacing of (111) and (002) planes are measured to be 0.218 and 0.184 nm, respectively, which show contract upon alloying compared to d-spacing of (111)_Ag_ and (002)_Ag_. The contracted value is around 7.35% for them. Owing to smaller atomic radius of Cu atom (0.12 nm) than Ag atom (0.1445 nm), only when Cu atoms have solute into Ag cell and form Ag-Cu alloy the shrinkage of d-spacing is reasonable. Here, it is of note that TEM used in this work is well calculated, and the deviation is under 2%. Therefore, interference caused by TEM deviation to the conclusion has been excluded. In addition, HAADF result shown in Figure 1c displays that contrast of the particles is brighter than gap area between particles, demonstrating a higher atomic number *Z* for nanoparticles. The lower *Z* corresponding gap area then is attributed from Cu element. This is because *Z* of Cu (*Z* = 29) is smaller than Ag (*Z* = 47). Combining the amorphous state in gap area observed in Figure 1b, we can draw that Ag_50_Cu_50_ catalyst actually is Ag-Cu alloyed nanoparticles embedded in amorphous Cu film.

### Electrochemical performance

**Figure 2:**
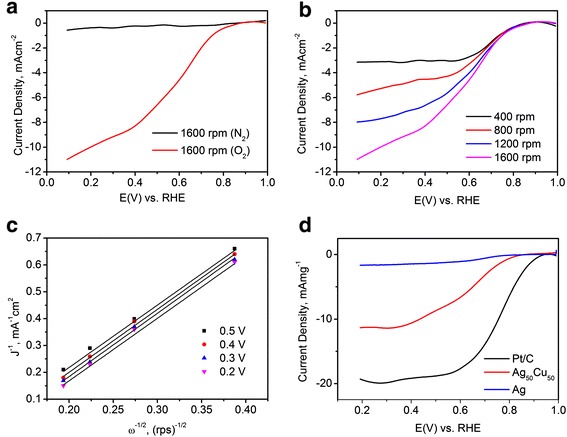
The electrochemical characterization on Ag_50_Cu_50_ catalyst. **(a)** The RDE curves of Ag_50_Cu_50_ catalyst in O_2_ and N_2_-saturated 0.1 M KOH solution; **(b)** the RDE curves at the rotation rates of 400, 800, 1,200, and 1,600 rpm; **(c)** the Koutecky-Levich plot of Ag_50_Cu_50_ catalyst; and **(d)** the ORR mass activity for Ag, Ag_50_Cu_50_, and Pt/C (20 wt%) catalysts.

A series of electrochemical characterizations have been carried out on Ag_50_Cu_50_ catalyst. Figure 2a shows RDE polarization curves of Ag_50_Cu_50_ catalyst with rotation rate 1,600 rpm in N_2_ and O_2_ saturated 0.1 M KOH solutions. It can be seen that there is reduction current density in O_2_-saturated KOH solution, while that in N_2_-saturated solution is flat and the value is close to 0 mA cm^−2^. The fact indicates that the catalyst indeed works on O_2_. Figure 2b shows a set of RDE curves with rotation rates of 400, 800, 1,200, and 1,600 rpm. The curves demonstrate that the current density increases with elevating rotation rate. It is because the higher speed of rotating disk electrode, the shorter diffusion distance of oxygen to catalyst surface. The Koutecky-Levich plots were then obtained from the limiting current density, as shown in Figure 2c. The plots show the inverse current density (*J*^−1^) as a function of ω^–1/2^. The detailed Koutecky-Levich equation [37] is expressed as follows:

1 $$ {J}^{-1}={J}_k^{-1}+{\left(0.62{nFCD}^{\frac{2}{3}}{V}^{-\frac{1}{6}}{\omega}^{\frac{1}{2}}\right)}^{-1} $$

where *J* is current density, *J*_k_ is the kinetic current density of the ORR, *n* is the number of transferred electrons during ORR, *F* is the Faraday constant, **C** is the bulk concentration of O_2_, *D* is the diffusion coefficient of O_2_ in 0.1 M KOH solution, *V* is the kinetic viscosity of the electrolyte for 0.1 M KOH, and *ω* is the angular velocity of the disk. After input of these values into Equation 1, *n* was determined to be 3.76, 3.87, 3.85, and 3.97 when the potential was 0.5, 0.4, 0.3, and 0.2 V, respectively. The result points out that a four-electron pathway occurred, which is a more efficient than the two-electron pathway [38,39].

We also compare the polarization curve of Ag_50_Cu_50_ catalyst to commercial Pt/C (20 wt%) and Ag catalysts on a RDE in O_2_-saturated KOH solution at 1,600 rpm, as shown in Figure 2d. Here, Ag catalyst was obtained through the same PLD process with Ag_50_Cu_50_. The limiting current density of Ag catalyst for ORR is only −2 mA mg^−1^ at 0.2 V. For Ag_50_Cu_50_ catalyst, it displays a significant improvement. The onset potential and limiting current density for ORR are 0.81 V and −12.3 mA mg^−1^ at 0.2 V, respectively. Pt/C catalyst has also been tested and is shown with black curve. Although there is a difference between Pt/C catalyst and Ag_50_Cu_50_ catalyst, it is still believed that the catalyst has excellent catalytic activity for ORR. Moreover, the low cost makes it an excellent candidate to substitute precious Pt-based catalysts.

### Zinc-air battery performance

**Figure 3:**
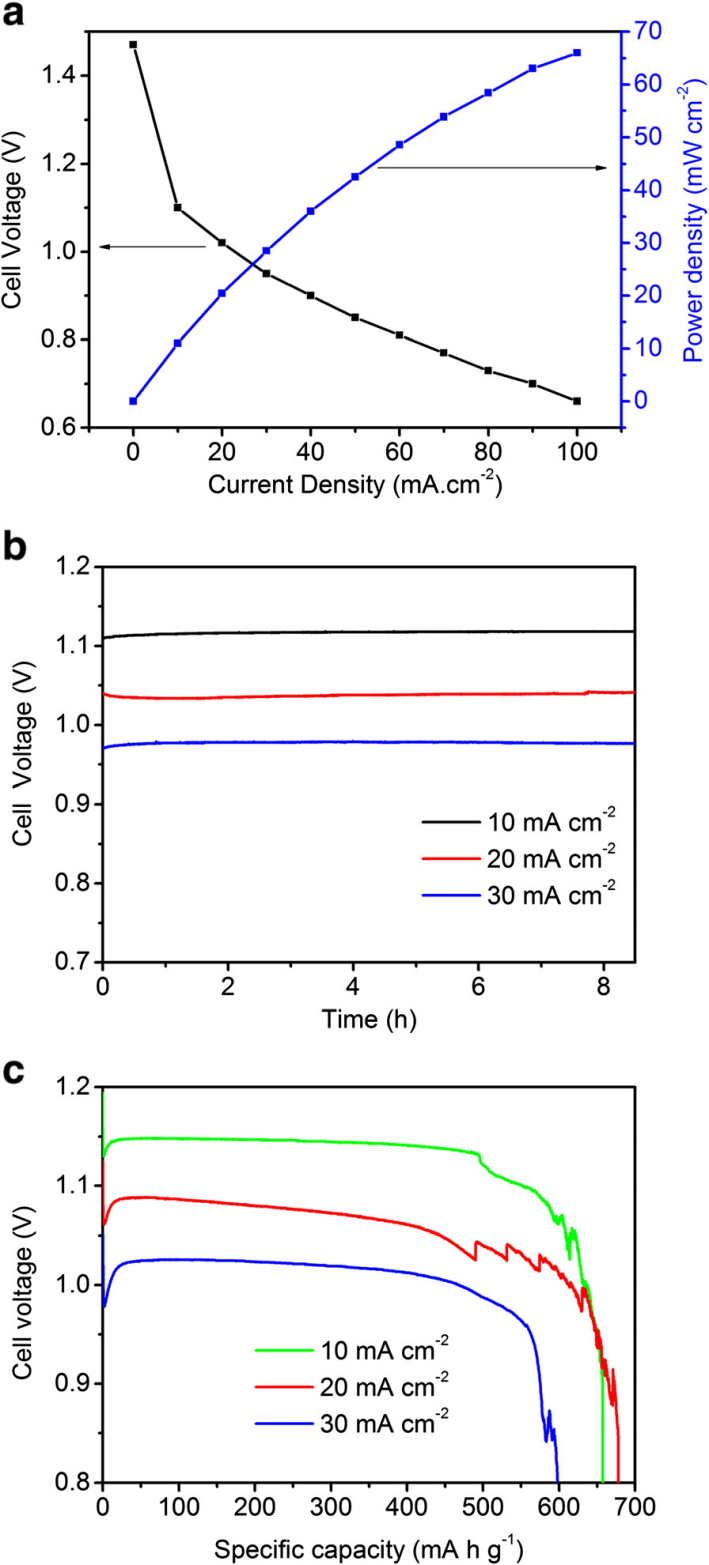
The performance of the primary zinc-air battery with Ag_50_Cu_50_ catalyst. **(a)** The cell voltage and power density curves of a primary zinc-air battery. **(b)** The cell voltage and time curves at different current density of 10, 20, and 30 mA cm^−2^ in the primary zinc-air battery. **(c)** The specific capacity curves of the primary zinc-air battery at 10, 20, and 30 mA cm^−2^. The electrolyte is 6 M KOH solution under natural air atmosphere.

Ag_50_Cu_50_ catalyst then was used in a cathode catalyst to evaluate its practical application in primary zinc-air battery. The results are shown in Figure 3. The cell voltage polarization and power density curves for the primary zinc-air battery are shown in Figure 3a. The cell voltage decreases with increasing current density, demonstrating that the cell performance shows a strong dependence on the resistance of the battery. The open circuit voltage of the cell is around 1.48 V closing to the theoretical value, and the maximum power density is 67 mW cm^−2^ at 100 mA cm^−2^. Figure 3b displays the change of cell voltage with time at a current density of 10, 20, and 30 mA cm^−2^. The initial discharge voltage for Ag_50_Cu_50_ catalyst decreases from 1.12 to 0.97 V when the current density within the range from 10 to 30 mA cm^−2^. The three curves keep flat after more than 8 h of discharge, indicating a high discharge voltage stability. As shown in Figure 3c, when the current density is 30 mA cm^−2^, the specific capacity of Ag_50_Cu_50_ catalyst-based battery is 598 mAh g^−1^ as normalized by the mass of consumed Zn anode, corresponding to specific energy density about 610 mWh g^−1^. When changing the current density to 20 mA cm^−2^, the specific capacity and specific energy density increase to 678 mAh g^−1^ and 725.5 mWh g^−1^, respectively, which is comparable to CoO*x*/carbon nanotube-based zinc-air battery [40]. The green curve corresponding to 10 mA cm^−2^ shows a sudden falling. It may result from shedding Zn plate during discharge process. The above results indicate that Ag_50_Cu_50_ catalyst prepared *via* PLD is a highly efficient catalyst in primary zinc-air battery.

**Figure 4:**
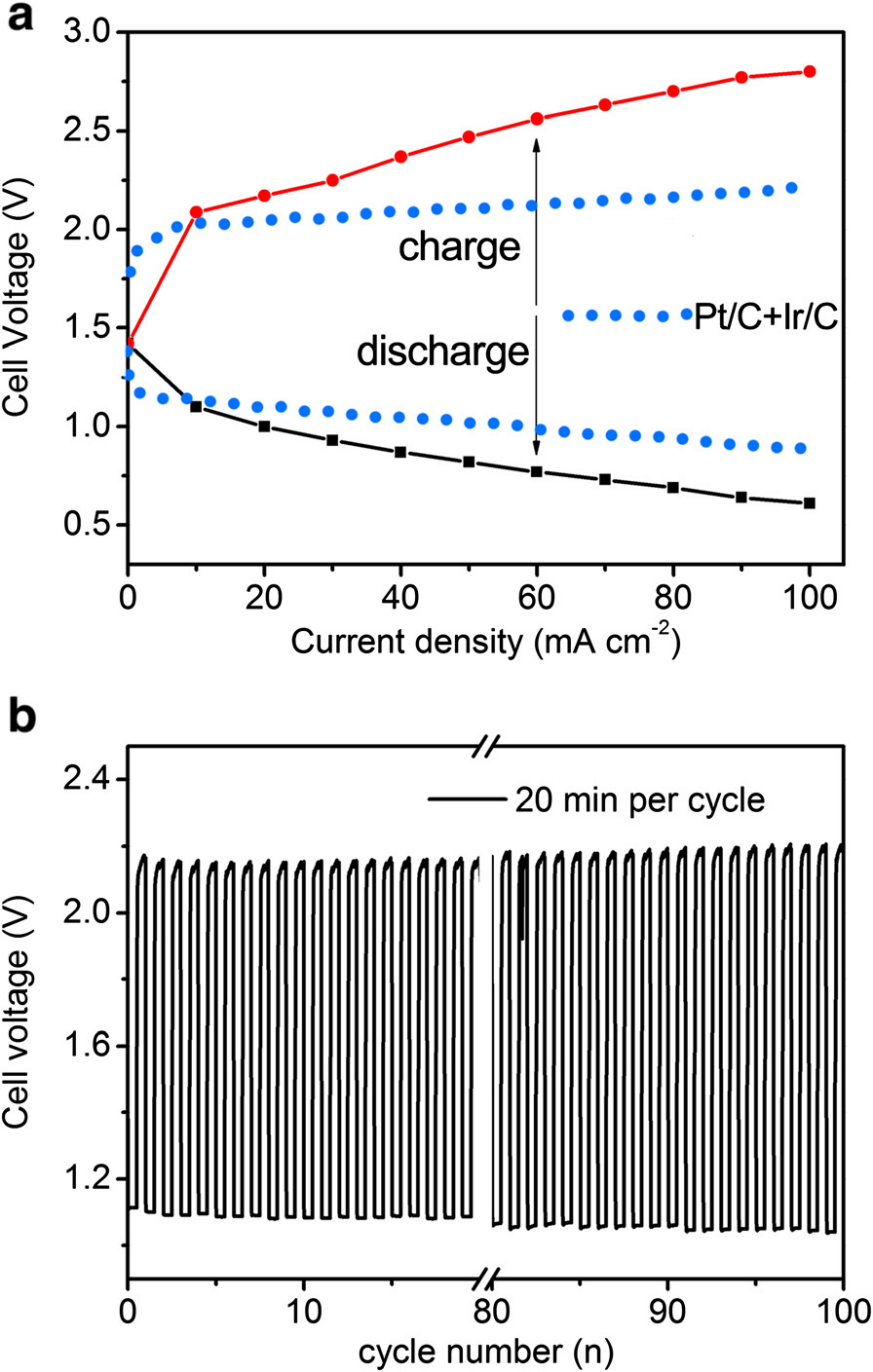
The performance of the rechargeable zinc-air battery with Ag_50_Cu_50_ catalyst. **(a)** Charge and discharge polarization curves of the rechargeable zinc-air battery using Ag_50_Cu_50_ catalyst compared with the one using Pt/C and Ir/C (blue dotted line). **(b)** The cycle performance of the rechargeable zinc-air battery with Ag_50_Cu_50_ catalyst. The electrolyte is 6 M KOH plus 0.2 M zinc acetate solution under natural air atmosphere.

Figure 4a shows the charge and discharge polarization curve of the resulting rechargeable zinc-air battery with Ag_50_Cu_50_ catalyst. The cell voltage rose with a steady trend from 1.4 to 2.7 V when the current density was increasing. When the current density was 20 mA cm^−2^, the cell voltages for charge and discharge were 1.0 and 2.1 V, respectively. Therefore, the over potential is 1.1 V, which is close to the value of Co_3_O_4_ nanodisk catalyst [41]. To well understand the performance of such battery, we also collect the polarization curve from commercial Pt/C and Ir/C catalyst-based zinc-air battery (blue dotted line) in reference [3]. It can be clearly seen that the over potential of Ag_50_Cu_50_ is approaching to Pt/C and Ir/C based battery at 20 mA cm^−2^. Someone may argue that 1.1 V is higher than reported non-precious catalyst-based batteries. The fact is the value was obtained from natural air atmosphere in this work, not the artificial high-density oxygen atmosphere. Therefore, the Ag_50_Cu_50_-based battery owns good practicability. The discharge cell voltage still keeps 0.7 V at a high current density of 100 mA cm^−2^, demonstrating an excellent battery performance. The cycle performance of the rechargeable battery is shown in Figure 4b. It underwent 100 cycles at 20 mA cm^−2^ with 20 min per cycle. The round-trip efficiency of the first cycle was 52.3%, and the last cycle was 50%. They do as well as that of zinc-air battery assembled with cobalt oxide-based cell [41,42], indicating a good stability for the Ag_50_Cu_50_ battery.

According to all the above electrochemical characterization and the battery performance, the excellent ORR catalytic activity is believed. It is speculated that these are resulting from the alloying between Ag and Cu. Before we verify the speculation, it is necessary to analyze the formation of AgCu alloyed film firstly. In general, whether a metal forms a continuous film or grows into discrete clusters (or nanoparticles) depends on the relative magnitudes of the adhesion energy (*E*_adh_) and the metal/vacuum interfacial free energy (*γ*_v/m_). If *E*_adh_ < 2*γ*_v/m_, three-dimensional growth is expected to occur *via* the Volmer-Weber growth mode [43,44]. As Ag satisfies the criterion [45], Ag nanoparticles have formed at the very beginning of PLD process, and they are showing as tiny amorphous particles at the beginning. In terms of Cu, it is speculated to form as amorphous Cu film using the criterion [46].

There are two possible ways for Cu atom solute into Ag cell. One is some Cu atoms may be surrounded inside the Ag nanoparticles during the PLD ejecting process. The second way is some Cu atoms included in the amorphous Cu film contacting surface of Ag nanoparticles. At this stage, Ag and Cu atoms mixed randomly within the tiny nanoparticles, and they have not formed the alloyed state. In Figure 4b, we can see that the amorphous particles are smaller than the crystallized ones in size. It indicates that they are in this stage.

After the above stage, the following are then the crystallization and growth for the Ag-rich amorphous nanoparticles. The substrate used in PLD is difficult to be definitely clear and pure, which consequently creates preferred sites promoting the nanoparticles to crystallize. In terms of growth, it may occur through coalescence, because of the relatively short diffusion distances [45]. Eventually, Cu atoms have solute into Ag cell *via* substituting some of Ag atoms and form into silver-rich AgCu solid solution nanoparticles, that is, AgCu alloyed nanoparticles. With increment of deposit time, the alloying degree between Ag and Cu elevates. However, if the deposit time is too long, AgCu alloyed nanoparticles may grow bigger and bigger, like the huge nanoparticles shown in Figure 4a. The ORR activity will get deteriorated, because the bigger the nanoparticles, the smaller the specific surface area.

According to the formation mechanism of AgCu film, the alloying between Ag and Cu is verified from the aspect of PLD process. In our previous work, it demonstrates that pure Cu material catalyzed ORR barely [8]. Therefore, the enhancement of ORR catalytic activity of Ag_50_Cu_50_ film actually is from AgCu alloyed nanoparticles. The synergistic effect between Ag and Cu within the alloyed nanoparticles calculated by simulation is verified through the experiments [47,33]. It is also consistent with the d-bond center theory [48,49]. The theory aims to design a highly active catalyst for ORR *via* synthesizing a binary or multi-alloy with various metals which have different absorption and desorption energy for oxygen [50]. Ag has the strong oxygen desorption energy, yet the Cu has forceful absorption energy. As a result, when Ag and Cu are formed into AgCu alloy or bimetallic nanomaterials, the product will improve the ORR activity to a great extent owing to the synergistic effect between Ag and Cu atoms.

In addition, the enhancement in ORR activity of Ag_50_Cu_50_ over Ag catalyst also can be explained by the ligand effect and strain effect. Regarding the ligand effect, it actually originates from the random Cu atoms solute into (near) surface of structure of various AgCu catalysts. In terms of the strain effect, it could be caused by the shorter Ag-Ag bond length in Ag_50_Cu_50_ catalyst than Ag catalyst, given the fact that d-spacing of Ag_50_Cu_50_ planes were smaller than the corresponding Ag planes. The two effects both improve the catalytic activity of the catalyst, which can weaken Ag-O binding energies and strengthen the O_2_ molecular adsorption to the surface of AgCu solid solution [51].

**Figure 5:**
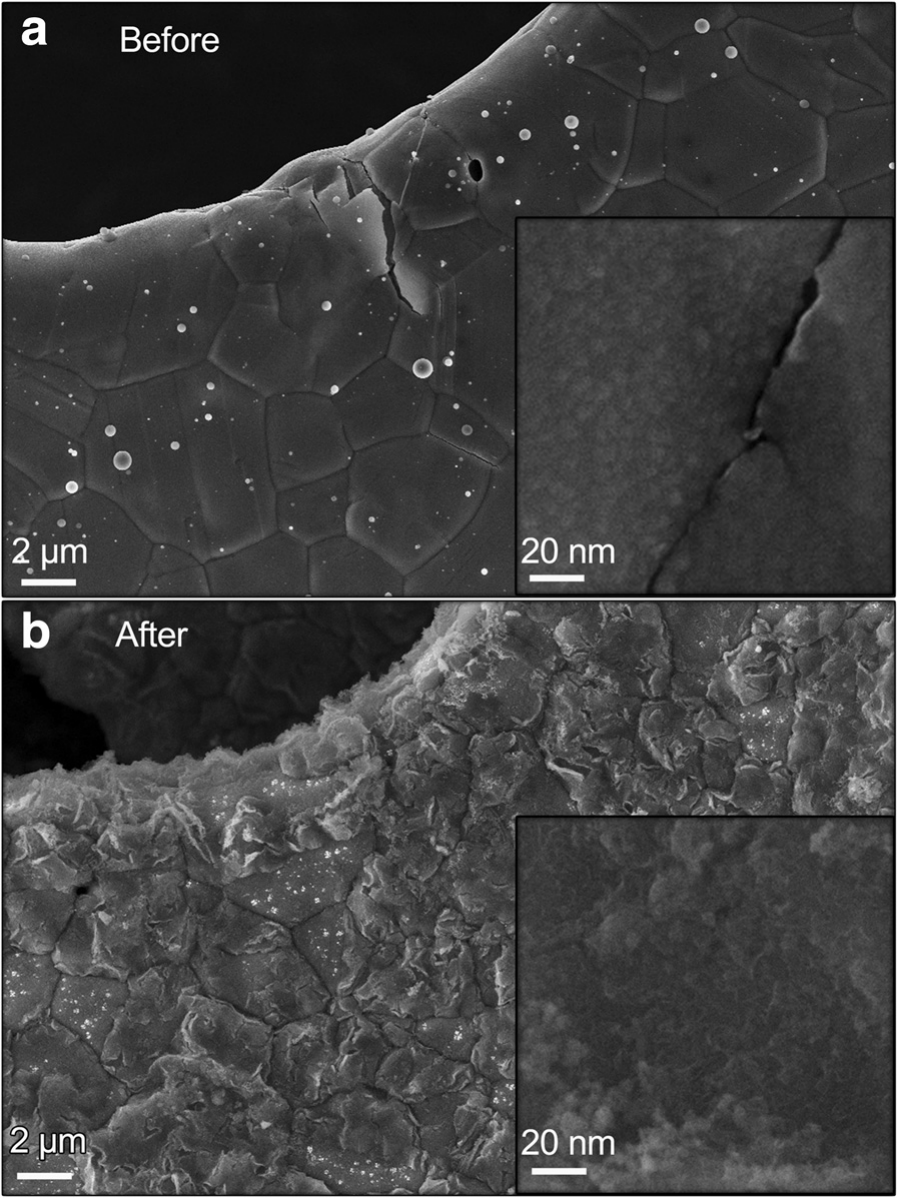
The morphology of the Ag_50_Cu_50_ catalyst before **(a)** and after **(b)** recycle performance test.

Since the good catalytic activity is attributed to alloyed AgCu nanoparticles, we can illustrate the good recycle stability of Ag_50_Cu_50_ catalyst-based zinc-air battery from the perspective of morphology. In Figure 5b, the morphology of Ag_50_Cu_50_ catalyst shows as flocculence after recycle test at a macro level, which is not as smooth as its original state shown in Figure 5a. From the inset image in Figure 5b, the particles which were previously embedded in Cu film escape from Cu film. Some particles may shed, while most of the particles expose more surface than before. Therefore, despite slight change of morphology, the retaining of AgCu alloyed nanoparticles and increased specific surface area help keep the good catalytic activity for Ag_50_Cu_50_ cell, even if it already underwent long charge and discharge recycles.

## Conclusions

We demonstrate a design of Ag-Cu film catalyst *via* PLD. The obtained Ag_50_Cu_50_ film shows excellent ORR catalytic activity. TEM results verify the catalyst actually is AgCu alloyed nanoparticles embedded in amorphous Cu film. The alloying between Ag and Cu from the embedded AgCu nanoparticles synergistically improves the ORR catalytic activity. Using the catalyst to assemble a zinc-air battery, the maximum power density is 67 mW cm^−2^ in primary zinc-air battery. The resulting rechargeable zinc-air battery shows low charge-discharge voltage polarization of 1.1 V at 20 mA cm^−2^ and high stability over 100 charge and discharge cycles in natural air.
